# Bile acids and gestational diabetes mellitus: exploring the link and implications - a review

**DOI:** 10.3389/fendo.2025.1574228

**Published:** 2025-07-10

**Authors:** Chunxia Lu, Changyi Li, Xiaoping Lei

**Affiliations:** ^1^ Department of Neonatology, Children’s Medical Center, The Affiliated Hospital of Southwest Medical University, Luzhou, Sichuan, China; ^2^ Sichuan Clinical Research Center for Birth Defects,The Affiliated Hospital of Southwest Medical University, Luzhou, Sichuan, China

**Keywords:** gestational diabetes mellitus, bile acids, glucose homeostasis, offspring, therapeutic applications

## Abstract

Gestational diabetes mellitus (GDM) represents a prevalent metabolic disorder related to pregnancy, posing significant risks to both the expecting mother and the developing fetus. Recent research indicates a potential connection between bile acids (BAs) and GDM, such as lithocholic acid (LCA), β-muricholic acid (β-MCA), and 6,7-diketolithocholic acid (6,7-diketoLCA), have been found to be significantly increased in GDM individuals, thereby with the potential to reveal their involvement in glucose metabolism and the underlying mechanisms of GDM development. Additionally, BAs have emerged as vital signaling molecules that regulate glucose and lipid metabolism by interacting with Farnesoid X receptor (FXR) and Takeda G protein-coupled receptor 5 (TGR5), highlighting their potential as novel therapeutic targets for GDM management. The aim of this manuscript is to comprehensively review the current understanding of the relationship between BAs and GDM, delving into their potential mechanistic roles, diagnostic significance, and possible therapeutic applications.

## 1 Introduction

Gestational diabetes mellitus (GDM) classically denotes abnormal glucose tolerance that manifests or is first identified during pregnancy, featuring glycemia and insulin disorders (1). The worldwide prevalence of GDM is escalating at a rapid pace (2). This condition is not only linked to adverse perinatal outcomes (3) but also increases women’s long-term risk of developing type 2 diabetes mellitus (T2DM) (4, 5) and metabolic syndrome (5, 6). Moreover, children born to mothers with GDM face a heightened risk of obesity, metabolic syndrome, future type diabetes (7), and brain development issues (8–10).

In recent years, numerous studies have delved deeper into the etiology and pathophysiology of GDM (11, 12). Emerging research has revealed a potential connection between bile acids (BAs) and GDM (13, 14) recently. BAs, amphipathic molecules synthesized in the liver from cholesterol and forming a crucial bile component (15), have traditionally been recognized for their role in the digestion and absorption of dietary fats (16). However, modern perspectives view BAs as more versatile molecules with diverse functions (17), including promoting intestinal epithelial regeneration (18, 19), regulating gene expression (20, 21), influencing insulin secretion (22, 23), epigenetic mechanisms (24, 25), fibrogenesis (26), lipid metabolism (27)and glucose metabolism (28). Consequently, alterations in BAs are strongly linked to metabolic disorders.

## 2 Bile acid metabolism

BAs encompass both primary and secondary types, as outlined in **Table 1**. The biosynthesis of BAs commences with the formation of primary BAs, predominantly in the liver. This process involves a sequence of 17 enzymes, including cytochrome p450, which alter the steroid ring of cholesterol. These enzymes eliminate the short aliphatic side chain and conjugate it primarily with glycine (75%) and taurine (25%). The end result is the conjugated primary BAs, specifically cholic acid (CA) and chenodeoxycholic acid (CDCA) (29, 30). Secondary BAs come into being through enzymatic modification of primary BAs by colon-dwelling bacteria, which utilize them as substrates for microbial metabolism (31). The BA pool, encompassing all BAs circulating within the enterohepatic circulation, comprises BAs present in the intestine (~85%–90%), gallbladder (~10%–15%), and liver (<1%) (32). The ratio of glycine (G)- to taurine (T)-conjugated BAs stands at approximately 3 to 1, establishing a hydrophobic pool (32).

**Table 1:** The classification of bile acids.

	Unconjugated BAs	Conjugated BAs	
		+Taurine	+Glycine
primary BAs	CA CDCA	TCA TCDCA	GCA GCDCA
secondary BAs	DCA LCA UDCA HDCA	TDCA TLCA TUDCA THDCA	GDCA GLCA GUDCA GHDCA

BAs, bile acids; CA, cholic acid; CDCA, chenodeoxycholic acid; TCA, taurocholic acid; TCDCA, taurocholic acid; GCA, glycocholic acid; GCDCA, glycochenodeoxycholic acid; DCA, deoxycholic acid; LCA, lithocholic acid; UDCA, ursodeoxycholic acid; HDCA, hyodeoxycholic acid; TDCA, taurodeoxycholic acid; TLCA, taurolithocholic acid; TUDCA, tauroursodeoxycholic acid; THDCA, taurohyodeoxycholic acid; GDCA, glycodeoxycholic acid; GLCA, glycolithocholic acid; GUDCA, glycoursodeoxycholic acid; GHDCA, glycohyodeoxycholic acid.

**Figure 1:**
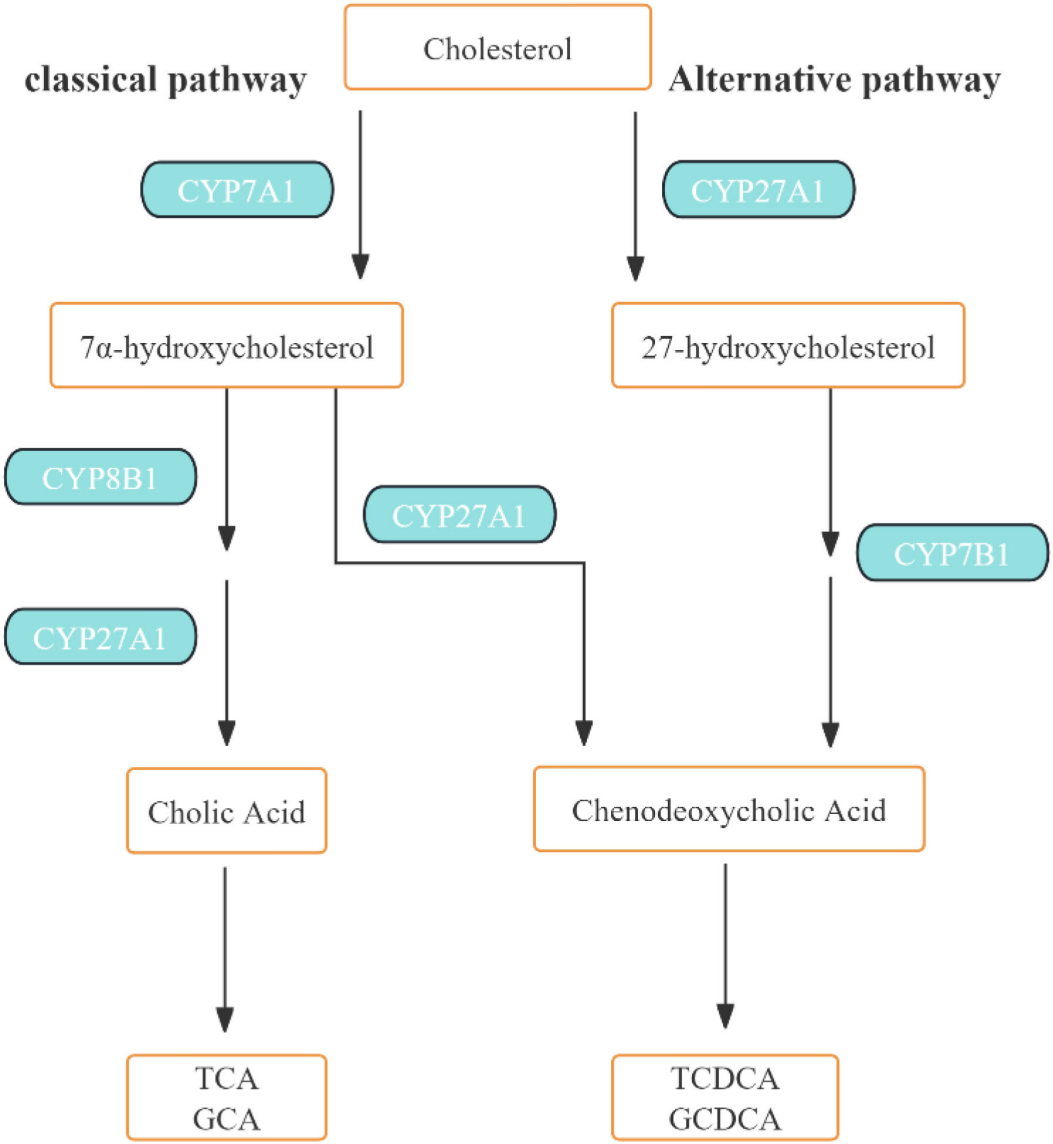
Bile Acid metabolism in liver. In the liver, cholesterol 7α-hydroxylase (CYP7A1) initiates the classical bile acid synthesis pathway by hydroxylation of the steroid rings at 7α-C for further modifications of the steroid rings, followed by steroid side chain oxidation and cleavage, whereas sterol 27-hydroxylase (CYP27A1) initiates the alternative bile acid synthesis pathway by oxidation of the steroid side chain followed by modifications of the steroid rings and cleavage of the side chain in the classic pathway. Cholic acid (CA) and chenodeoxycholic acid (CDCA) are the two major primary bile acids synthesized in the human liver.

The synthesis of BAs can commence via several routes (**Figure 1**). The classic pathway involves the metabolism of cholesterol 7α-hydroxylase (CYP7A1) to form 7α-hydroxycholesterol, which is subsequently hydroxylated by sterol 12α-hydroxylase (CYP8B1) or sterol 27-hydroxylase (CYP27A1). Alternatively, the second (or alternate) pathway sees the formation of 27-hydroxycholesterol from cholesterol via CYP27A1, followed by hydroxylation via oxysterol 7α-hydroxylase (CYP7B1) (30). A third pathway involves the oxidation of cholesterol to 24- and 25-hydroxycholesterol by cholesterol 24-hydroxylase (CYP46A1), an enzyme predominantly expressed in the brain (33).

## 3 Bile acids and glucose homeostasis

Recently, BAs have garnered attention due to their involvement in glucose metabolism and the secretion of glucoregulatory hormones (34, 35). Studies have shown that BAs regulate glucose homeostasis by directly interacting with the FXR (36) and the TGR5 (37, 38), or indirectly by promoting the synthesis of fibroblast growth factor 15 (FGF15) in the intestine, which is induced by FXR (39, 40). Specifically, certain BAs activate FXR in the intestine, triggering the production of FGF15/19 and enhancing the expression of pancreatic β cells (41). This mechanism exerts diverse effects on hepatic BA metabolism, lipid metabolism, protein metabolism, and glucose metabolism (42). Furthermore, BA-mediated TGR5 signaling boosts the release of intestinal glucagon-like peptide 1 (GLP-1), thereby increasing glucose-stimulated insulin secretion from pancreatic β cells (43). The receptors specific to BAs and the precise molecular mechanisms underlying their effects on glucose metabolism will be further explored (**Table 2**).

**Table 2:** Effects of BAs and receptors on glucose metabolism and mechanisms.

BA Receptors	Function	Mechanism	References
FXR	Regulating hepatic glucose production and reducing serum glucose levels	Suppression of gluconeogenic genes, due to FXR activation of the transcriptional repressor SHP	Ma et al. (36)
		Protection from skeletal muscle lipotoxicity and improvement of peripheral insulin sensitivity, via FXR-dependent liver lipid metabolism	Ma et al. (36)
		Reduced weight gain due to adipose tissue browning, downstream of FXR-dependent alterations in BA composition	Fang et al. (44)
		Increased GLP-1 and insulin secretion, due to shifts in gut bacteria composition, which increase the TGR5 agonist TLCA	Pathak et al. (45)
		Increased secretion of FGF15 and/or FGF19, thereby repressing gluconeogenesis, and increasing glycogen synthesis and energy expenditure	Kir et al. (42); Potthoff et al. (46); Renga et al. (47)
		Expressed in human pancreatic β-cells and stimulates insulin gene transcription producing a positive control on glucose dependent insulin secretion	Renga et al. (47)
TGR5	TGR5 has a protective role in glucose homeostasis	TGR5 activation in enteroendocrine cells increases the release of GLP-1 which maintains homeostasis of blood glucose by promoting glucose-induced insulin secretion, suppressing glucagon release, delaying gastric emptying, promoting satiety, and increasing glucose disposal in the peripheral tissues	Cao et al. (48); Kuhre et al. (49); Lasalle et al. (50)
FGF15 and/or FGF19	Maintaining normoglycemia	Reduced hepatic gluconeogenesis, downstream of FGF15- and/or FGF19-dependent dephosphorylation of the gluconeogenic transcription factor CREB	Potthoff et al. (46)
		Increased hepatic glycogen synthesis, due to FGF15-/FGF19-dependent activation of an ERK-GSK3α/β phosphorylation cascade	Kir et al. (42)
		Reduced body weight and adiposity	Lan et al. (51)

BAs, bile acids; FXR, farnesoid X receptor; SHP, small heterodimer partner; GLP-1, glucagon like peptide 1; TGR5, takeda G-protein receptor 5; TLCA, taurolithocholic acid; FGF15, fibroblast growth factor 15; FGF19, fibroblast growth factor 19; CREB, Cyclic AMP-regulatory element-binding protein; ERK, extracellular signal–regulated protein kinase; GSK3α/β, glycogen synthase kinase 3α and 3β.

Given that different BAs exhibit unique affinities for FXR and TGR5, and they play varying roles in glucose metabolism, it becomes imperative to investigate whether the BA profile undergoes changes in patients with GDM. Determining the clinical significance of any such alterations is also crucial.

## 4 Bile acids in pregnant women with GDM and their offspring

The quantity of BAs differed significantly between mothers with GDM and those without. A study revealed that pregnant women with GDM had higher serum total bile acid (TBA) levels than their non-GDM counterparts during the first trimester (52). Notably, elevated serum TBA concentrations during pregnancy have been positively correlated with an augmented risk of GDM (13, 14).Although a causal relationship between GDM and serum TBA levels has not been conclusively established, it is apparent that GDM is often associated with higher serum TBA levels. Additionally, when maternal serum TBA levels surpass 40 mmol/L, the likelihood of fetal complications increases by 1%-2% for every additional mmol/L (53). Consequently, we postulate that altered serum TBA could be a potential influencing factor in the relationship between GDM and complications in offspring. However, some studies found no significant differences in TBA levels between GDM and non-GDM groups when measured in the second or third trimester (54). The substantial heterogeneity observed across studies, primarily attributable to variations in the timing of TBA measurement, suggests that the relationship between TBA levels and GDM is not straightforward. This indicates that the role of TBA as a biomarker for GDM may be highly sensitive to the gestational period during which it is measured (55).

Pregnant women with GDM not only encounter elevated serum TBA levels, but also demonstrate alterations in their BA profiles when compared to those without GDM. Research indicates that, in GDM pregnancies, serum concentrations of glycodeoxycholic acid (GDCA), taurodeoxycholic acid (TDCA), CA, dehydro-lithocholic acid (dehydro-LCA), and iso-deoxycholic acid (iso-DCA) are notably diminished (56). Conversely, certain BAs, such as glycohyodeoxycholic acid (GHDCA), taurohyodeoxycholic acid (THDCA), hyodeoxycholic acid (HDCA), LCA, β-MCA, and 6,7-diketoLCA, have been found to be significantly increased in GDM individuals (56). In summary, the modifications in BAs associated with GDM are intricate, underscoring the importance of understanding these changes to gain further insight into GDM.

Although numerous studies have established that the serum BA profiles of mothers with GDM undergo changes, the impact on fetal/neonatal serum BA profiles remains unclear. Recent studies have indicated that a higher prevalence of GDM among women with intrahepatic cholestasis of pregnancy (ICP), offering potential insights into this issue (57, 58). Previous studies have revealed that umbilical cord from ICP pregnancies exhibits elevated levels of CDCA, CA and LCA compared to controls (59). Based on these findings, we hypothesize that variations in BAs among GDM mothers may also lead to alterations in BA metabolism in their offspring. Furthermore, a study has documented significant changes in BA metabolism within the amniotic fluid (AF) during the second trimester of GDM-diagnosed pregnancies (60). Given that the AF primarily consists of fetal urine, this study lends credence to our hypothesis. However, direct evidence remains lacking and further investigation is warranted to elucidate the specific changes occurring.

## 5 Predictive value of BAs in GDM

Currently, the oral glucose tolerance test (OGTT) is widely regarded as the gold standard for diagnosing GDM (1). However, it is important to note that OGTT typically diagnoses GDM between 24–28 gestational weeks. By this time, irreversible fetal changes, such as epigenetic modifications (61), may have already occurred. Therefore, the identification of early predictors would be beneficial in improving the management of GDM and minimizing adverse outcomes for both the mother and the fetus.

ICP, characterized by elevated TBA levels, is strongly associated with an increased vulnerability to GDM (57, 58). This suggests a potential link between BA changes and the development of GDM. Based on this, we hypothesize that BAs could serve as valuable biomarkers for GDM diagnosis and risk stratification. Indeed, studies have shown that pregnant women with higher serum TBA levels during the first to second trimester face an increased risk of developing GDM. This indicates that TBA may represent a new risk factor for GDM (13), likely due to its correlation with insulin sensitivity (62). However, it’s worth noting that Zhu et al. have found TBA levels to remain stable in the GDM group when compared to those with normal glucose tolerance (63). This discrepancy could be partially attributed to methodological differences, specifically the distinction between TBA measured by enzymatic cycling assay and individual BAs detected via mass spectrometry (MS). This finding underscores the importance of focusing on individual BA components related to glucose metabolism.

Individual BAs have emerged as promising biomarkers for the diagnosis and risk stratification of GDM (**Table 3**). Gao et al. have specifically highlighted β-MCA as a potential biomarker that can distinguish between GDM patients and healthy controls (54). Notably, β-MCA levels are elevated in GDM patients, possibly due to enhanced α-muricholic acid (α-MCA) C7-isomerase activity. This activity subsequently leads to increases in terminal GHDCA and THDCA levels through specific metabolic channels (54). GDCA, on the other hand, shows a significant decline in GDM patients. Its level is inversely correlated with insulin sensitivity and positively correlated with β-cell compensation, making it a valuable biomarker candidate for assessing these factors (63). Van Nierop et al. have indeed linked GDCA to insulin secretion and resistance, with increased GDCA triggering insulin secretion in a GLP-1-dependent manner (66). This explains why, despite an elevation in GDCA levels after glucose intake in GDM patients, the lower baseline GDCA levels are insufficient to promote insulin secretion via GLP-1, ultimately leading to glycemic dysregulation. Importantly, these markers have been identified post-diagnosis, and further studies are warranted to determine if they are altered in early pregnancy serum samples of women with GDM.

**Table 3:** The predictive value of BAs in GDM.

Predictive markers	The association with GDM risk	References
β-MCA	positive	Gao et al. (54)
GDCA	negative	Zhu et al. (63)
TCA	positive	Wu et al. (64)
LCA	negative	Wu et al. (64)
GUDCA	Negative (≤ 0.07 nmol/mL)	Li et al. (65)
DCA	Negative (≤ 0.28 nmol/mL)	Li et al. (65)

BAs, bile acids; GDM, gestational diabetes mellitus; β-MCA, β-muricholic acid; GDCA, glycodeoxycholic acid; TCA, taurocholic acid; LCA, lithocholic acid; GUDCA, glycoursodeoxycholic acid; DCA, deoxycholic acid.

Recent evidence also suggests that BAs could serve as early diagnostic marker for GDM. Circulating BAs levels during early pregnancy are associated with GDM risk. Specifically, taurocholic acid (TCA) is positively, while LCA negatively associated with GDM risk (64). Additionally, low serum levels of glycoursodeoxycholic acid (GUDCA) and deoxycholic acid (DCA) during early pregnancy are independently linked to an increased risk of GDM development (65). Secondary BAs are converted from primary BAs by gut microbiota (22), and an abnormal gut microbiome may reduce this conversion, particularly of GUDCA and DCA, which may contribute to the etiology of GDM. Furthermore, in a rodent model, an elevated serum CA concentration, coupled with reduced BA receptors, such as FXR and TGR5, is associated with GDM (67). Therefore, further validating the diagnostic value of these BA metabolites in the early stages of GDM through animal experiments holds significant promise for early and timely intervention in GDM, potentially reducing poor outcomes.

## 6 Potential values for BA intervention in the GDM

The treatment of GDM primarily aims to normalize hyperglycemia and mitigate the risk of unfavorable pregnancy outcomes. A crucial aspect of GDM management involves lifestyle interventions, such as dietary adjustments, physical activity, and weight control. If glycemic targets are not achieved through these interventions, it is necessary to introduce glucose-lowering pharmacologic therapy (68, 69). Although these treatments offer short-term benefits, their long-term effects on children exposed to antidiabetic medication during pregnancy remain uncertain. Hence, there is an urgent need for therapies that can improve both maternal and fetal glucose metabolism. BAs have emerged as vital signaling molecules that regulate glucose and lipid metabolism by interacting with FXR and TGR5 receptors (70–73). This suggests that therapeutic approaches targeting BAs could potentially be a powerful new strategy for GDM management.

The FXR agonist obeticholic acid (OCA) has been found to improve dyslipidemia and reduces the impact of pregnancy on insulin resistance in a mouse model of GDM, although it does not affect glucose tolerance (74). However, the limited effects of OCA in pregnant mice indicate that its agonistic action alone may not fully counteract the metabolic consequences of reduced FXR activity during pregnancy. Therefore, when considering FXR agonists for treating metabolic disorders during pregnancy, it is essential to consider the potential inhibition of FXR activity during gestation to ensure the safety of the pharmaceutical agent.

Studies have indicated that lower levels of GDCA are associated with increased risk of adverse pregnancy outcomes in GDM patients (63). Based on this, we hypothesize that GDCA supplementation may reduce these adverse outcomes, but further research is required to validate this hypothesis. Notably, UDCA has been shown to significantly lower fasting plasma glucose, hemoglobin A1c (HbA1c), and insulin concentrations, indicating a beneficial effect on glucose homeostasis (75). Preliminary data from studies involving UDCA treatment in women with ICP also suggest a reduction in insulin resistance (76). The study emphasizes that UDCA’s potential as an effective therapy for improving maternal glycemia in GDM. Although direct evidence supporting UDCA’s use in GDM treatment is lacking, some trial protocols have been designed (77), paving the way for future studies. Furthermore, animal studies have provided additional insights. For instance, mice fed a high-fat diet (HFD) exhibit elevated fasting glucose and a reduced BA pool size, but supplementation with CA improves insulin resistance (78). Another study found that secondary BAs exert a protective effect on pancreatic islet β-cells in diabetic rats (79).

BA sequestrants, which effectively disrupt the enterohepatic circulation of BAs and significantly reduce plasma cholesterol levels, provide evidence for a connection between BA and glucose metabolism (80). Numerous lipid-lowering studies have demonstrated that BA sequestrants, exemplified by colesevelam hydrochloride (81), cholestyramine (82) and colestilan (83), can also decrease plasma glucose and glycosylated hemoglobin levels. This suggests a potential role for these agents in the treatment of T2DM. Given the application of BAs in managing T2DM, it is reasonable to postulate that BAs may also hold promise in treating GDM. However, direct evidence supporting this hypothesis is currently lacking. Thus, further exploration into the therapeutic benefits of BA metabolites for GDM is crucial. While BA sequestrants demonstrate proven efficacy in T2DM management through TGR5/GLP-1 pathway (84, 85), their application in pregnancy warrants meticulous investigation. The placental transfer potential of BA sequestrants derivatives and their effects on fetal BA circulation remain undefined. The present study indicates that the use of BA sequestrants can impede the absorption of fat-soluble vitamins, such as vitamin K, potentially increasing the risk of neonatal cerebral bleeding (86), emphasizing the need for trimester-specific therapeutic development.

In summary, a novel approach to the treatment of GDM with BA has demonstrated significant potential. Evidently, future research should be directed towards three primary areas: first, conducting research on longitudinal BA profiling; second, performing randomized controlled trials (RCTs) of BA modulators; and third, investigating microbiome - BA interactions.
